# Factors associated with early return visits to the emergency department in patients with vaso-occlusive crisis

**DOI:** 10.1186/s12873-025-01192-1

**Published:** 2025-03-01

**Authors:** Mohammed Khalid Alageel, Hassan Mohammad Aloraini, Alanoud Mansour Alessa, Alanoud Binmethem, Ghada Alsaleh, Sarah Abdullah Almubrik, Abdulaziz Alalshaikh, Kholood K. Altassan

**Affiliations:** 1 Department of Emergency Medicine, College of Medicine, King Saud University, Riyadh, KSA Saudi Arabia; 2 Department of Emergency Medicine, University of British Columbia, Vancouver, Canada; 3 Global Center for Mass Gathering Medicine, Ministry of Health, Riyadh, KSA Saudi Arabia; 4 Department of Family and Community Medicine, College of Medicine, King Saud University, Riyadh, KSA Saudi Arabia

**Keywords:** Emergency department, Hemoglobinopathy, Saudi Arabia, Vaso-occlusive crisis, Sickle cell disease, Early return visit

## Abstract

**Background and aim:**

One of the most common presentations of sickle cell disease (SCD) in the emergency department (ED) is acute severe pain episodes due to a vaso-occlusive crisis (VOC). Management of these episodes is primarily through intravenous pain control, but patients often return to the ED with the same complaint a few days after discharge. While some global studies have explored the risk factors for ED revisits due to VOC, the literature is lacking in the adult population, specifically in Saudi Arabia where SCD prevalence is high. The goal of this study is to measure the incidence of ED 72-hour early revisit (ERV) among SCD patients due to a VOC episode and to identify factors that might be associated with an ERV in this population. We conducted a retrospective cohort study using the electronic medical records, retrieving all patients who presented to the ED with a VOC from the period of 2017 to 2022.

**Results:**

This study included 120 VOC visits. The percentage of 72-hour ERV to the ED among VOC patients was 39.2%, in which 91.5% received opioids, and 31.9% were admitted during the return visit. Return visitors’ median age was 29, most of them were male. There was no statistically significant correlation found between the patients’ 72-hour ERV to the ED and their age, gender, comorbidities, history of exchange transfusion, pain score, or dose of opiates received. Of the variables measured at the index visit only the direct bilirubin level, and time to first opioid dose was associated with 72-hour ERV with an OR of 1.08 (95%CI: 1.0 to 1.16, *P* = 0.022) and 0.99 (95%CI: 0.99 to 0.99, *P* = 0.012) respectively.

**Conclusion:**

We found that 39.2% of VOC episodes discharged from the ED had an ERV. This rate is higher than what is reported internationally. Additionally, the lack of clear predictors for revisits raises doubts regarding the efficacy of the ED ‘’treat and release’’ approach in this population.

**Clinical trial number:**

Not applicable.

**Supplementary Information:**

The online version contains supplementary material available at 10.1186/s12873-025-01192-1.

## Introduction

Sickle Cell Disease (SCD) is an autosomal recessive hereditary disorder of hemoglobin, characterized by abnormally shaped sickle red blood cells [1, 2]. It is among the most common hematological conditions in Saudi Arabia, with a prevalence reaching up to 2.6% in certain regions [3].

The most common manifestation of SCD is vaso-occlusive crisis (VOC). It occurs due to an acute occlusion of the small blood vessels caused by the distorted morphology and increased adhesiveness of the sickled erythrocytes [4]. VOC usually presents with sudden, often symmetrical pain, most commonly involving the lower back, legs, abdomen, and knees [5, 6]. VOC is the most common cause of emergency department (ED) visits and hospitalization among SCD patients [7–9]. Local evidence suggests that in Saudi Arabia, six out of ten SCD patients report visiting the ED at least three times every 6 months due to VOC episodes [10]. In addition to the debilitating pain experienced by SCD patients during episodes, VOCs can also lead to complications such as hepatic and renal injury, acute chest syndrome, cerebrovascular accidents, and multi-organ failure [11]. These potentially life-threatening consequences result in substantial morbidity and early mortality in this population [12]. In addition, poor patient outcomes and increased costs have been demonstrated as a result of the associated recurrent ED visits.

Patients who suffer from VOC episodes present with variable severity, with some requiring more frequent ED visits and revisits as well as multiple admissions. However, the factors associated with episode severity, frequency and outcomes are still unclear. Some studies have suggested concomitant comorbidities increase the likelihood of VOC episodes [12]. Other studies looking at the impact of hematological variables on VOC revisit rates demonstrated inconsistent results, with some finding no associations [13], while others highlighted variables such as white blood count (WBC) and blood bilirubin as possible markers of disease severity [14]. The literature has also suggested that biomarkers such as lactate dehydrogenase (LDH) are predictive of VOC-associated inpatient outcomes [15]. Some researchers have further proposed that care received during the ED visit, specifically regarding prescription practices, plays an important role in outcomes, with higher opioid administration during the visit and lack of oral opioid dispensing upon discharge predicting early revisits [16, 17].

EDs treat patients with acute severe pain due to a variety of conditions (renal colic, headache, abdominal pain, etc.), including VOC, which often require intravenous (IV) analgesic medications [18]. Discharging a patient with inadequate pain control can potentially contribute to the chronification of pain [19]. When patients return to the emergency department (ED) after being assessed, treated, and discharged, with the same or a related complaint, it is labeled an early return visit (ERV). These patients may not have received the appropriate treatment or diagnosis [20]. The time frame used to define an ERV varies but is most often defined as within 72 h of the index visit [21].

The literature on ED ERVs, specifically among SCD patients with VOC, is inconsistent, with no consensus on the timeframe that constitutes an ERV and wide variation on the frequency of occurrence. A US study in 2014 that looked at 72-hour ERVs in the pediatric ED found SCD patients had the highest rate of revisit among all patients at 10.7% [22]. A more recent multicenter study in 2023 reported the rate of ERV in the same population to be 16.7% [23]. A study conducted in New York found that among 1456 pediatric and adult VOC patients who visited the ED, 35.3% revisited within 30 days [16]. Another US based study, where they established a day hospital as an alternative to the ED for adult management of VOC, showed that 9.5% of discharges resulted in a three day ERV to either the ED or the day hospital [24]. However, research on 72-hour ED ERVs rates among adult VOC patients is lacking. There are also no studies exploring this topic regionally or locally in Saudi Arabia.

The aims of this paper are (1) to measure the incidence of ED ERVs within 72 h among SCD patients with a VOC episode in Riyadh, Saudi Arabia, and (2) to identify factors that might be associated with an ERV in this population.

To our knowledge, this is the first attempt to measure the frequency of ERVs among VOC patients in Saudi Arabia and to explore the associated risk factors.

## Methodology

### Study design and setting

This is a retrospective cohort study set in King Khalid University Hospital (KKUH) using data provided by the medical records department. KKUH is a large academic tertiary care hospital in Riyadh, Saudi Arabia. The facility’s ED receives around 100,000 visits per year. It provides comprehensive medical care, including hematology and oncology services.

### Selection of participants (inclusion/exclusion criteria)

We selected all SCD adult patients who presented to the ED with an episode of VOC between 2017 and 2022. Data was extracted using the International Classification of Diseases (ICD-10) codes. We received the data from the IT department in an Excel sheet format that included the patient’s medical registration number, chief complaint with the ICD code, date and time of the visit, length of stay, discharge date and time. Individual visit data was manually reviewed to verify the diagnosis. Our case eligibility criteria were SCD patients 14 years or older (based on the institutional definition for “adult patients”) who had a diagnosis of VOC on their initial visit to the ED (the index visit), and were discharged by the ED physician or the inpatient consulting team from the ED. Patients who required admission, left prior to completion of assessment, left against medical advice, or refused to be admitted during the index visit were excluded. To prevent the inclusion of patients with an unresolved ongoing VOC episode, we also excluded patients who had a documented admission or ED visit one week prior to their index visit. In addition, some patients were excluded as they were triaged out based on the institution’s policy of having minimal pain and a low Canadian Triage and Acuity Scale (CTAS) score. We define an ERV as returning to the ED after discharge from the index visit due to persistent VOC symptoms within a 72-hour period. Patients who were discharged and did not meet the criteria for an ERV were analyzed for comparison.

### Outcome measures

The outcome measure is the rate of 72-hour ERVs to the ED among adult SCD patients presenting with VOC. We collected data on several patient and ED visit characteristics to include in the analysis. Patient variables (age, gender, comorbidities, history of exchange transfusion, compliance on hydroxyurea), index visit variables (type of opioids, time to first dose, total dose, pain score, length of stay, opioids prescription upon discharge, lab variables, number of ED visits, number of ED admissions), and revisit variables (length of stay, admission at revisit, opioids administration). Given that a variety of opioids can be given in the ED, in order to standardize the dosing we used opioid equivalent dosing [25].

### Statistical analysis

Data was analyzed using SPSS version 28 (IBM Co., Armonk, NY, USA. Relevant descriptive data were reported. Numerical data were presented as median and interquartile range (IQR) and were analyzed by Mann Whitney-test. Categorical data were presented as frequency and percentage (%) and analyzed using the Chi-square test or Fisher’s exact test when appropriate. Simple and multiple logistic regression models were performed to assess the association between different factors and 72-hour early revisit to the ED. A two tailed *p*-value of < 0.05 was considered statistically significant.

All data were collected after obtaining the approval of the Institutional Review Board of King Saud University (reference no. E-22-6922). Consent was deemed not applicable by the IRB due to retrospective nature of data extracted from the hospital health information system.

## Results

**Fig. 1:**
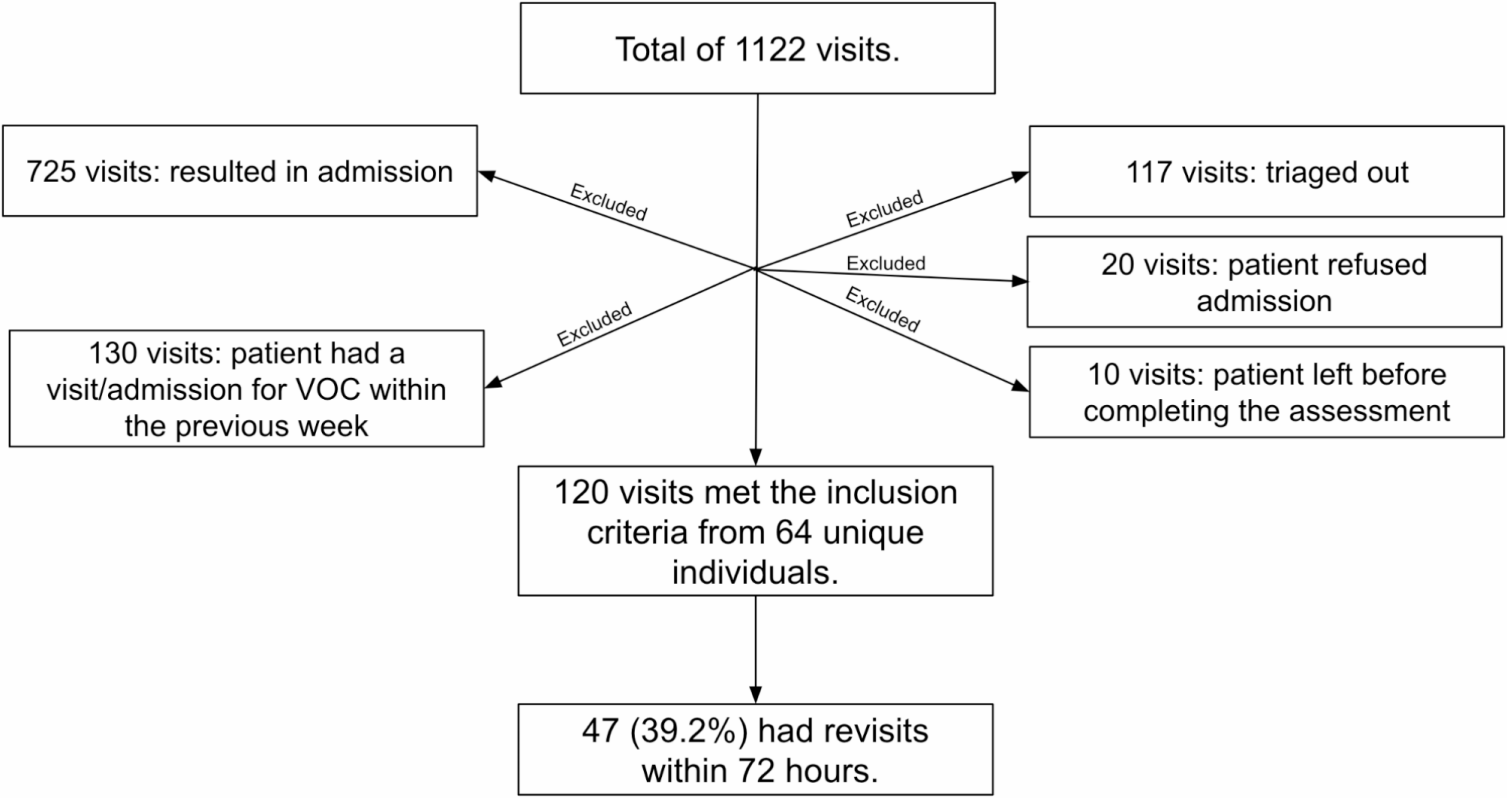
Flowchart describing exclusion criteria and final sample

Between 2017 and 2022, a total of 1122 visits were made to the KKUH ED with a documented diagnosis of VOC. After applying our previously specified exclusion criteria, the remaining number of eligible visits for inclusion in this study was 120, which were made by 64 unique individuals (Fig. 1). We will henceforth refer to these visits as the index visit.

Among the 64 patients responsible for the 120 index visits, most were male (58.3%), and the median age was 28 years (IQR 22–34.75). More than 40% of the visitors reported having comorbidities; specifically, 46 out of 120 had biliary disease and 3 had type II diabetes. Less than half of the visits (43.3%) were by patients who had previously had a documented transfusion exchange. Although nearly all the visits were made by patients who had previously been prescribed Hydroxyurea (92.5%, *n* = 111), half were non-compliant (50.8%, *n* = 61). A large majority of the patients in this sample were administered opioids during their index ED visit (90.8%, *n* = 109). Everyone who was administered opioids received morphine, and in 6 cases the patients also received tramadol and fentanyl. One case was given ketamine. Table 1 summarizes the patient characteristics and features of the care received at the index visits for the 120 visits. Additionally, S1 describes the characteristics of the 64 unique patients and is provided in the supplementary materials.

**Table 1:** Characteristics of the index VOC visits

		Total visits (*n* = 120)
Age (years)	Median (IQR)	28 (22–34.75)
	Range	14–50
Gender	Male	70 (58.3%)
	Female	50 (41.7%)
Comorbidities	No Comorbidities	71 (59.2%)
	Biliary disease	46 (38.3%)
	Diabetes mellitus II	3 (2.5%)
History of transfusion exchange	Yes	52 (43.3%)
Compliant on hydroxyurea	Yes	50 (41.7%)
	No	61 (50.8%)
	Not on hydroxyurea	9 (7.5%)
Opioid administration	Yes	109 (90.8%)

Among the 120 visits, we found that the ERV rate within 72 h was 39.2% (47 visits), a third of which (15 visits) required admission for further care and pain control.

Comparing the patient characteristics of those who had a 72 h return visit and those who did not, we found that while the median age of patients was similar, there was a larger proportion of male patients among those who had a return visit. We also found that both groups had a comparable history of transfusion exchange, with approximately 43% prevalence in each.

Clinical features at the index visit such as pain score, history of ED visits and previous hospital admissions via the ED were slightly higher among those who had a 72-hour ERV revisit compared to those who did not. Although the dosage of administered opioids at the index visit was the same for both groups, a slightly smaller proportion of those who later had an ERV were administered opioids in the ED. Additionally, the median time to administration was 26 min shorter for this group. Furthermore, only 40% of those who did return to the ED were discharged on opioids, compared to more than 50% among those who did not.

Analysis of both groups’ laboratory investigations during the index visit demonstrated almost no difference in levels of white blood count (WBC), hemoglobin (Hb), neutrophil count, mean corpuscular volume (MCV) and platelet count. Median lactate dehydrogenase (LDH) levels were 9% higher for patients who had a 72 h return visit, while median levels of total bilirubin and direct bilirubin were 16.2% and 14.4% higher for this group, respectively. Table 2 summarizes these findings.

**Table 2:** Relationship between patients’ characteristics, index visit assessment, laboratory investigations of patients’ index ED visit and their 72 h. Return to ED

	72 h ERV to the ED		*p*-value
	No (*n*** = 73)**	Yes (*n*** = 47)**	
***Patient characteristics***			
Age (years)	28 (21–35)	29 (22–34)	0.605
Gender			
Male	41 (56.2%)	29 (61.7%)	0.548
Female	32 (43.8%)	18 (38.3%)	
Has comorbidities	26 (35.6%)	23 (48.9%)	0.147
History of transfusion exchange	32 (43.8%)	20 (42.6%)	0.89
***Hydroxyurea use***			
Not on hydroxyurea	6 (8.2%)	3 (6.4%)	0.649
On hydroxyurea - compliant	39 (53.4%)	22 (46.8%)	
On hydroxyurea - noncompliant	28 (38.4%)	22 (46.8%)	
***Index visit***			
Pain score	5.5 (4–8)	6 (5–8)	0.383
Opioids administered	67 (91.8%)	42 (89.4%)	0.75
Time to first opioid dose (minutes)	87 (34–180)	61 (20–150)	0.073
Opioid dose total (mg)	10 (5–15)	10 (5–15)	0.877
Discharge on opioids	38 (52.1%)	19 (40.4%)	0.213
Number of previous ED visits*	9 (4–21)	10 (7–32)	0.245
Number of previous admissions*	5 (2–15)	6 (3–16)	0.548
***Laboratory investigations***			
WBC (x10^3^cells/µl)	13.35 (10.88–15.95)	13.3 (10.18–16.71)	0.917
Hb (g/dL)	9 (8.08–10.13)	9.35 (8.38–10.83)	0.181
Neutrophil (x10^3^cells/µl)	7.25 (5.63–9.63)	7.2 (5.23–9.7)	0.934
MCV (fL)	90.3 (82.93–95.75)	91.1 (83.95–95.05)	0.582
PLT (x10^3^cells/µl)	412.5 (309.25–517)	416.5 (263.25–480)	0.451
LDH (IU/L)	429 (373–600)	467.5 (308.75–573.75)	0.829
Total bilirubin (µmol/L)	40.85 (29.91–66.08)	47.45 (36.95–75.47)	0.195
Direct bilirubin (µmol/L)	7.58 (5.74–10.59)	8.67 (6.28–13.05)	0.11

Data are presented as median (IQR) or frequency (%) as appropriate. Statistical significance at *p*-value < 0.05. ERV: Early return visit, ED: Emergency department * For VOC

In a univariate analysis we found time to first opioid administration to be the only index visit characteristic significantly associated with the rate of 72-hour ERV (OR = 0.99. *P*-value = 0.015). We selected the variables that were associated with ERVs at *p*-values less than 0.2 to analyze in a multivariate model. Thus, in addition to the time to first opioid dose, we also included hemoglobin level (OR = 1.21, *p*-value = 0.1), direct bilirubin level (OR = 1.05, *p*-value = 0.078), and having comorbidities (OR = 1.73, *p*-value = 0.149) in the model. After adjustment in the multivariate model, we found only time to first opioid dose and direct bilirubin were significantly associated with rate of ERV with an OR of 0.99 and 1.07 respectively. [Table 3]

**Table 3:** Logistic regression model for factors associated with 72 h. Return to ED among

	Univariate			Multivariable		
	uOR	95%CI	*P* value	aOR	95%CI	*P* value
**Age (years)**	1	0.96 to 1.04	0.927	-	-	-
**Sex**						
Male	Ref					
Female	0.80	0.38 to 1.68	0.548	-	-	-
**Has Comorbidities**	1.73	0.82 to 3.65	0.149	1.41	0.57 to 3.52	0.459
**Has History of transfusion exchange**	0.95	0.45 to 1.99	0.890	-	-	-
Compliant on Hydroxyurea*	0.77	0.37 to 1.60	0.480	-	-	-
**Number of ED visits**						
1–10	Ref					
11–20	0.98	0.37 to 2.57	0.959	-	-	-
> 20	1.2	0.51 to 2.82	0.681	-	-	-
**Previous admissions**						
0	Ref					
1–3	0.88	0.21 to 3.64	0.854	-	-	-
4–10	1.58	0.39 to 6.28	0.520	-	-	-
> 10	1.02	0.25 to 4.12	0.977	-	-	-
**Opioid given**	0.75	0.22 to 2.62	0.655	-	-	-
**Time to first opioid dose (minutes)**	0.99	0.99 to 0.99	0.015	0.99	0.99 to 0.99	0.012
**WBC (x10** ^**3**^ **cells/µl)**	1.01	0.93 to 1.09	0.843	-	-	-
**Hb (g/dL)**	1.21	0.96 to 1.52	0.100	1.30	0.99 to 1.71	0.061
**MCV (fL)**	1	0.97 to 1.03	0.905	-	-	-
**PLT (x10** ^**3**^ **cells/µl)**	0.99	0.99 to 1.00	0.464	-	-	-
**Total bilirubin (µmol/L)**	1	0.99 to 1.02	0.402	-	-	-
**Direct bilirubin (µmol/L)**	1.05	0.99 to 1.11	0.078	1.07	1.01 to 1.15	0.021

uOR: Unadjusted odds ratio, aOR: Adjusted odds ratio, CI: Confidence interval, Statistical significance at *P* value < 0.05. *Includes self-reported non-compliant and not prescribed hydroxyurea

## Discussion

In our study we found the local incidence of treated VOC cases with a 72-hour ERV to the ED to be 39.2%, which is much higher than rates reported in other studies. Benjamin et al. [24] estimated the 72-hour ERV amongst VOC cases to be 9.5% of adult patients treated for uncomplicated VOC. The lower rate may be attributed to various factors, such as the studied sample being predominantly from an outpatient day hospital, and thus subjects have milder cases of VOC and less severe baseline SCD disease. In contrast, our findings are comparable to the 30-day ERV rate of 35.3% reported by Glassberg et al. [16]. The high incidence of ERVs in this study, which is more than three times the rates elsewhere for the same duration [24], is striking. We pose several potential factors that may have led to this finding. To start, in this study we focused only on adult ED patients in our cohort. One study looking at the demographics of SCD pediatric patients found that older patients had greater odds of having ERVs and reported the 16–18 age group having the highest number of ERVs [23]. This suggests disease progression with age, requiring more ED visits, which has previously been observed [16]. Another factor could be the predominant SCD genotypes in our population. A previous study investigating hematological disease genotypes in a similar sample of patients from the same hospital demonstrated that almost all patients having HbSS, HbS thalassemia, which manifest more severe disease and more frequent VOCs episodes [26–28]. Further the rate of patients regularly taking oral hydroxyurea was low, which has been shown to directly affect frequency and severity of VOC episodes [29].

In contrast the 30-day readmission rate for admitted patients treated for VOCs reported by Ballas et al. was 50% and the 7-day readmission rate was 16%. This was attributed to premature discharge from hospital with the episodes of pain not fully controlled [30]. A recent Saudi study, also conducted at King Khalid University Hospital, demonstrated comparable rates with 58% of SCD VOC patients requiring readmission within 30 days and 32% within the first week [26].

This is the first study to attempt to quantify the rate of ERV due to VOC in Saudi Arabia. First, we note that the reported number of VOC episodes amongst patients with SCD in the literature varies widely. A recent systematic review of 52 papers found a frequency of more than three incidents of VOC per year ranging between 4 and 67%. This significant variation is likely due to differences in defining an episode of VOC, with episodes presumed to be under-reported. There is also variation in severity of baseline disease in the studied populations [26]. Local studies on SCD patients found the incidence of more than three VOC episodes per year to be 39.4% [13]. 

The significance of ED ERVs is the subject of much research, as it is often used as a metric of the care provided in the ED. Despite the heterogeneity of definitions and cut offs used to define an ERV, most of the literature on the topic reports the overall ERV rate for the same complaint to be less than 10% [31–33]. Local data from the KKUH quality unit indicates an overall 72-hour ERV rate of 2% for all presentations during the same study period. ERVs have been attributed to inadequacies of care provided during the initial visit, as well as patient specific characteristics [34]. Additionally, they have been implicated as risk factors for adverse events such as ICU admissions and mortality, often resulting in increased resource utilization [33, 35].

It is worth noting that from the sample studied we excluded patients who were admitted to the hospital or had an ED visit within one week prior to the index visit for a VOC. This was done to ensure a washout period, removing the possibility of ongoing VOC episodes being included more than once. This is based on pathophysiological data suggesting that VOC episodes in most patients could last up to a week [5, 30]. Further this would minimize the inclusion of patients that cycled through the emergency department with multiple visits for the same episode of VOC but would request to be discharged from the ED rather than be admitted to the hospital despite the inpatient physician’s recommendation.

We analyzed several variables previously reported in the literature as risk factors for VOC ERVS. Amongst the analyzed factors we identified both a hematological and a care related variable as significantly associated with ERVs. Our analysis showed that the increase in direct bilirubin was associated with increased ERVs, although total bilirubin had no statistically significant impact. This may be a result of the small sample size in this study as total bilirubin, which increases with hemolysis, may theoretically be a marker of SCD activity. Further, similar to other hematological variables, such as white blood cell count (WBC) and absolute neutrophil count, total bilirubin has been linked to frequency of visits for SCD [14]. We also report the time to the first opioid dose as a significant factor for ERV for VOC. Our results show that shorter time to administration of the first dose of opioids is associated with higher rate of ERVs. Although more severe presentations in the ED would induce quicker administration of pain control, this finding is less likely to reflect severity of the VOC presentations, as the observed pain scores did not differ between those who had an ERV and those who did not. The correlation may be an artifact of frequent visitors avoiding ED peak times and visiting during less busy periods to minimize wait times. Pain scores during the ED visit have been identified as possible risk factors for ERVs in previous literature [7, 30]. As well as the total dose of opioids administered during the visit [16, 17].

Another factor that has been implicated in increasing ERVs is having a history of multiple admissions. Zakaria et al. attempted to identify hematological and clinical factors predicting VOC episodes, and only found having a history of more than three admissions as a risk factor for frequent VOC [13].

To address high ERV rates, our hospital established a Frequent Visitor Program (FVP), designed to create structured ED treatment plans and ensure multidisciplinary follow-up for patients with frequent visits, including those with VOC. This approach, supported by studies such as Glassberg et al. and Lalloo et al., has been shown to reduce ED utilization and improve outcomes by promoting individualized care, better pain management, and increased compliance with therapies like hydroxyurea [36, 37]. 

Conventional textbook and guidelines-based care suggests that VOC cases may be managed in the ED and discharged when pain episodes appear controlled [38, 39]. Our findings suggest that the “treat and release” strategy for adult VOC patients has a substantial failure rate and VOC cases should not be approached like other acute pain presentations in the ED, as there are no consistently identified risk factors. This is further supported by the high readmission rates of patients suffering from VOCs in the literature. Potential measures that may help in mitigating the issue include providing close follow up for all VOC patients treated and discharged from the ED, implementing short stay units that allow admission for all VOC patients, and offering all VOC presenting patients an admission.

We note several potential limitations in this study. First, the retrospective approach to data collection and reliance on electronic records can result in misclassification and missing potential cases due to failed index visit coding. However, we attempted to minimize this impact by manually checking for miscoded prior visits. Second, we could not account for ERVs that took place outside of the studied site, which likely suggests our ERV rate may be an underestimation. Additionally, our study period included the COVID-19 pandemic period, where lockdowns were in place. Although the institution only limited patients based on CTAS score during that period and was still receiving patients with severe pain throughout, this still may have impacted the number of SCD patients seeking medical care. Further, our findings may have been biased by the triaging out of ED patients who were classified as non-emergent using the CTAS. This may have resulted in the selection of patients with more severe presentations. Another important limitation is the small sample size in this study. This likely influenced our ability to detect any statically significant factors related to the rate of ERV. Finally, the single center design limits the generalizability of our findings.

We recommend future multi-center prospective studies which include recording indicators of baseline SCD disease severity. Observational or interventional designs that compare different treatment strategies for VOC in the ED, such as ‘’treat and release’’, in-patient, and short stay would also help identify the most effective approach to limit ERVs while minimizing financial and social impact on both the patient and health care system.

In summary, the rate of ERV in the ED among VOC patients in our cohort was 39.2%, which is notably higher than what has been reported in the literature in other locales, with no clearly identified risk factors. These findings question the effectiveness of a “treat and release” strategy conventionally recommended for adult patients suffering from a VOC.

## Electronic supplementary material

Below is the link to the electronic supplementary material.

Supplementary Material 1

## Data Availability

The entire de identified dataset, data dictionary and analytic code for this investigation are available upon request, from the date of article publication by contacting Doctor Alageel, MD, FRCPC at email: plasma45@mail.ubc.ca.
