# Supplementary material for: Factors associated with early return visits to the emergency department in patients with vaso-occlusive crisis
Source: BMC Emerg Med. 2025 Mar 1;25:33. doi: 10.1186/s12873-025-01192-1 (PMC11872308; doi:10.1186/s12873-025-01192-1)
Supplement: Supplementary file 1 — Supplementary Material 1 [file 12873_2025_1192_MOESM1_ESM.docx]

**Supplementary materials**

**S1: Characteristics of the patients responsible for the VOC index visits**

|  |  | **Total patients**  **(n = 64)** |
| --- | --- | --- |
| **Age (years)** | **Median (IQR)** | 28 (19.75 – 34) |
|  | **Range** | 14 - 50 |
| **Gender** | **Male** | 33 (51.6%) |
|  | **Female** | 31 (48.4%) |
| **Number of index visits** | **Median (IQR)** | 1 (1 - 2) |
|  | **Range** | 1 - 10 |
| **Comorbidities** | **No Comorbidities** | 46 (71%) |
|  | **Biliary disease** | 16 (25%) |
|  | **Diabetes mellitus II** | 2 (3.1%) |
| **History of transfusion exchange** | **Yes** | 21 (32.8%) |
| **Compliant on hydroxyurea** | **Yes** | 21 (32.8%) |
|  | **No** | 35 (54.7%) |
|  | **Not on hydroxyurea** | 8 (12.5%) |
